# The Effect of Egg Consumption in Hyperlipidemic Subjects during Treatment with Lipid-Lowering Drugs

**DOI:** 10.1155/2012/672720

**Published:** 2012-06-24

**Authors:** Theerawut Klangjareonchai, Supanee Putadechakum, Piyamitr Sritara, Chulaporn Roongpisuthipong

**Affiliations:** ^1^Department of Medicine, Faculty of Medicine, Ramathibodi Hospital, Mahidol University, Bangkok 10400, Thailand; ^2^Research Center, Faculty of Medicine, Ramathibodi Hospital, Mahidol University, Bangkok 10400, Thailand

## Abstract

*Background*. Limiting egg consumption to avoid high cholesterolemia is recommended to reduce risk of cardiovascular disease. However, recent evidences suggest that cholesterol from diet has limited influence on serum cholesterol. *Objective*. To assess the effect of egg consumption on lipid profiles in hyperlipidemic adults treated with lipid-lowering drugs. *Material and Method*. Sixty hyperlipidemic subjects, mean age of 61 years, who had been treated with lipid-lowering drugs. Every subject was assigned to consume additional 3 eggs per day with their regular diet for 12 weeks. Measurements for lipid profiles and body compositions were performed. *Results*. An additional consumption of 3 eggs per day for 12 weeks increased HDL-cholesterol by 2.46 ± 6.81 mg/dL (*P* < 0.01) and decreased LDL-cholesterol to HDL-cholesterol ratio by 0.13 ± 0.46 (*P* < 0.05). No significant changes were found in other lipid profiles. Body weight and body mass index were significantly increased at 12th week by 0.52 ± 1.83 kg and 0.31 ± 0.99 kg/m^2^, respectively (*P* < 0.05). *Conclusion*. In hyperlipidemic adults who were treated with lipid-lowering drugs, the consumption of additional 3 eggs per day to their regular diet will increase the level of HDL-cholesterol and decrease the ratio of LDL-cholesterol to HDL-cholesterol.

## 1. Introduction

Cardiovascular disease (CVD) is still a major public health problem and a leading cause of death in modern world, even in Thailand [1]. Because elevated low-density lipoprotein cholesterol (LDL-C) has been identified as a major risk factor for CVD, dietary guidelines to prevent CVD emphasize on reduction of dietary cholesterol intake to less than 300 mg/day for healthy adults or less than 200 mg/day for persons with elevated cholesterol or heart disease [2]. Egg yolk is a major source of dietary cholesterol with an average of 200 mg cholesterol in one egg [3]. On the other hand, egg is also an inexpensive complete food with low-calorie, high-quality protein sources as well as other important nutrients, such as minerals, folate, and vitamins B [4, 5]. In comparing with other animal protein sources, egg contains proportionately less saturated fat, which is a strong dietary determinant of elevated LDL-C and increased risk for CVD [6]. 

Clinical trials in children, younger adults, and the elderly showed increase in both LDL-C and HDL-C with no alteration in the LDL-C to HDL-C ratio after egg consumption [7–9]. A study in 40 hyperlipidemic adults who had not been treated with lipid-lowering drugs revealed that 2 eggs consumption per day for 6 weeks were not detrimental to endothelial function and serum lipids in hyperlipidemic subjects [10].

To the best of our knowledge, no previous report on the effect of egg in hyperlipidemic adults during treatment with lipid-lowering drugs. Therefore, we try to investigate the relationship between additional 3 eggs consumption per day in these subjects with lipid profiles. 

## 2. Subjects and Methods

Sixty subjects (58 women and 2 men) who had been diagnosed for hyperlipidemia and treated with lipid-lowering drugs with stable lipid profiles for 12 weeks were recruited. The participants who have been diagnosed as having egg allergy, hypothyroidism, coronary disease, diabetes mellitus, liver, or kidney problems were excluded from the study. An additional exclusion criterion was the regular use of hormone replacement therapy. 

All subjects did not change the dosage of lipid-lowering drugs during the 12 weeks of the study. All subjects were assigned to consume additional 3 eggs per day for 12 weeks and recorded amount of eggs that they consumed everyday. Subjects were not provided any other foods apart from eggs for their additional diet. Moreover energy intake was not restricted. Subjects were asked to maintain their normal routine physical activity during this study. The weight, height, body mass index, blood pressure, body composition, serum lipid profiles, 4-day dietary record, and daily egg record were performed and collected every 4-week interval. The biochemical tests including plasma glucose, renal and liver function tests, and hematological parameters were measured before and after the study.

The study was reviewed and approved by the Committee on Human Research of Ramathibodi Hospital, Mahidol University. The subjects were informed in detail about the study and informed consents were obtained from all of them.

## 3. Dietary Assessment

 Subjects completed detailed 4-day dietary records. They were required to maintain the food records for 3 weekdays and 1 weekend day. The interviewers collected the data and estimated the serving portions with the use of household measuring cups, spoons, and ruler to assist the subjects in the recall of foods eaten. During the study, the subjects were asked to check on a daily egg record sheet. 

## 4. Lipid Profile

The serum lipid profiles included total cholesterol (TC), triglyceride (TG), high-density lipoprotein cholesterol (HDL-C), and low-density lipoprotein cholesterol (LDL-C) which were measured by enzymatic methods (Boehringer Mannheim Corporation, Mannheim, Germany). 

## 5. Body Composition

Body composition was determined with the use of the InBody 720 body composition analyzer (Biospace Corporation, Seoul, Republic of Korea). Body composition was measured in the morning after an overnight fast. Body mass was recorded to the nearest 100 gram on a calibrated digital scale. Analyses were performed by the same technician who was unaware of the study details. 

## 6. Statistical Analysis

Baseline characteristics and serum lipid profiles of all subjects were reported by using mean ± standard deviation (SD). Statistical analysis was conducted using SPSS software version 13.0 for windows. All outcome measurements among baseline and each period data were assessed using two-way repeated measures ANOVA. *P* < 0.05 was considered statistically significant.

## 7. Results

Sixty hyperlipidemic subjects participated in this study. Ninety-six point six percent of the subjects were female. The age of the subjects was 45 to 85 years, with a mean age of 61.8 years (Table 1). Two subjects dropped out because of being unable to complete 12-week program, and one subject dropped out because of moving to another province. All subjects did not smoke or drink alcohol. The compliance to egg consumption between 4th, 8th, and 12th week were 96.0%, 98.8%, and 98.9%, respectively. Lipid profiles during 12 weeks prior to the beginning of the study did not show significant change when compared to baseline.

Body weight and body mass index were significantly increased at the 12th week. Body fat weight, lean body weight, and total body water did not show significant change as well (Table 2).

 Additional consumption of 3 eggs per day for 12 weeks increased HDL-C by 2.46 ± 6.81 mg/dL (*P* < 0.01) and decreased LDL-C to HDL-C ratio by 0.13 ± 0.46 (*P* < 0.01). The study showed no significant change of TG (*P* = 0.97), TC (*P* = 0.62), and LDL-C (*P* = 0.59) (Table 3). The biochemical tests of plasma glucose, renal and liver function tests, and hematological test did not significantly change before and after the study.

## 8. Discussion

The results of this study showed that egg consumption did not unfavorably influence serum cholesterol or other measurement of the lipid profile, in a previously noninvestigated population of hyperlipidemic adults who were treated with lipid-lowering drugs. In addition, egg consumption led to an increase in HDL-C as well as a decrease in LDL-C to HDL-C ratio, the major determinants of CVD risk. 

The other cohort study that has specifically examined the relationship between egg consumption and CVD included 37,851 men and 80,002 women who were free from chronic diseases of the Health Professionals Follow-Up Study and the Nurses' Health Study. After adjusting for multiple confounders, those eating >7 eggs/week (as compared with those consuming <1 egg/week) had no increased risk of CVD or stroke in either healthy men or women [11]. In two other cohorts, eating eggs more frequently was not associated with an increase in stroke or CVD incidence [4, 12].

Clinical trials conducted in healthy postmenopausal women and men aged 60 years or older have clearly demonstrated that women and men classified as hyporesponders to dietary cholesterol had no changes in LDL-C and HDL-C after consuming 3 eggs per day for 4 and 12 weeks, respectively. In contrast, those individuals who were classified as hyperresponder exhibit increases in both LDL-C and HDL-C with the result of no changes in the LDL-C to HDL-C ratio [13, 14]. In hyperlipidemic adults, 2 eggs consumption daily for 6 weeks had no adverse effect on endothelial function and serum cholesterol or other measures of the lipid profiles compared to sausage and cheese consumption [10].

The lipid profile after consuming additional 3 eggs per day in hyperlipidemic adults, who were treated with lipid-lowering drugs, changed similar to those of hypo-responders: no change in LDL-C, increase in HDL-C, and decrease in LDL-C to HDL-C ratio. These findings in keeping with a previous study of the effect of egg consumption in patients on HMG-CoA reductase inhibitor showed significant increases in HDL-C in subjects given 2 or 4 eggs daily [15]. The absence of an increase in plasma cholesterol in hyporesponders may be explained by their ability to maintain cholesterol homeostasis by decreasing the absorption of dietary cholesterol or suppressing endogenous synthesis [13]. Lipid-lowering drug in our study is HMG-CoA reductase inhibitor which can inhibit endogenous cholesterol synthesis like hypo-responders [16]. Increasing in HDL-C level by 1 mg/dL may reduce the risk of CVD by 2 to 3 percent [17], and decreasing in LDL-C to HDL-C ratio by 1 unit may decrease the risk of myocardial infarction by 50 percent [18]. Moreover, daily intake of 3 eggs significantly increases adiponectin that is both anti-inflammatory and antiatherogenic hormone [19]. In this study, HDL-C significantly increased while LDL-C to HDL-C ratio significantly decreased after egg consumption which may provide protection against development of CVD. Egg consumption failed to show the association to LDL-C and TC, but egg consumption showed the results with parameters to reduce risk of CVD. 

## 9. Conclusion

The results of this study suggest that an addition of 3 eggs per day to regular diet can raise the level of HDL-C and decrease the ratio of LDL-C to HDL-C in hyperlipidemic adults who are treated with lipid-lowering drugs. The results from this study will provide added-value knowledge for simple understanding among general population about egg consumption, especially that egg cholesterol is not equal to blood cholesterol. Further testing in individuals with established CVD is now justified to clarify the place of eggs in a judicious and heart-healthy diet.

## Figures and Tables

**Table 1:** Baseline clinical characteristics of study population.

Variable	Values
Sex female : male	58 : 2
Age (year)	61.8 ± 8.3
Systolic blood pressure (mmHg)	127.9 ± 9.5
Diastolic blood pressure (mmHg)	75.6 ± 9.7
Body weight (kg)	60.0 ± 7.7
Body mass index (kg/m^2^)	25.4 ± 3.3
Lipid lowering drugs	
Rosuvastatin 5 mg/day	17 (28.3%)
Atorvastatin 10 mg/day	12 (20.0%)
Simvastatin 20 mg/day	31 (51.7%)

Values are mean ± SD except otherwise stated.

**Table 2:** Mean ± SD of the body weight, BMI, and body composition.

	Baseline	4th week	8th week	12th week
Body weight (kg)	60.0 ± 7.7	60.3 ± 8.1	60.3 ± 8.1	60.5 ± 8.1*
Body mass index (kg/m^2^)	25.4 ± 3.3	25.7 ± 3.5	25.6 ± 3.4	25.8 ± 3.4*
Lean body weight (kg)	40.9 ± 4.8	41.0 ± 4.8	40.5 ± 5.0	40.6 ± 5.2
Lean body (%)	68.7 ± 6.1	68.3 ± 5.6	67.4 ± 5.2	67.4 ± 5.5
Body fat weight (kg)	19.0 ± 5.3	19.4 ± 5.3	19.8 ± 4.9	19.9 ± 4.9
Body fat (%)	31.3 ± 6.1	31.7 ± 5.6	32.6 ± 5.2	32.6 ± 5.5
Total body water (kg)	30.8 ± 3.1	30.9 ± 3.1	30.5 ± 3.2	30.6 ± 3.3
Total body water (%)	51.7 ± 4.8	51.5 ± 4.4	50.8 ± 3.9	50.8 ± 4.2

^∗^ *P* value <** **0.05 compared to baseline value.

**Table 3:** Mean ± SD of serum lipid profiles during the study.

	Baseline	4th week	8th week	12th week
TC (mg/dL)	193.4 ± 30.7	195.5 ± 27.7	194.1 ± 33.9	191.8 ± 28.9
TG (mg/dL)	109.5 ± 43.9	113.9 ± 47.4	116.7 ± 50.9	109.8 ± 42.4
HDL-C (mg/dL)	54.6 ± 12.5	53.9 ± 10.2	55.1 ± 11.3	57.0 ± 11.8**
LDL-C (mg/dL)	108.6 ± 28.5	108.5 ± 24.8	108.0 ± 27.8	106.9 ± 23.8
LDL-C : HDL-C	2.1 ± 0.6	2.1 ± 0.6	2.0 ± 0.6	1.9 ± 0.5*

^∗^ *P* value** **<** **0.05 compared to baseline value.

^∗∗^ *P* value** **<** **0.01 compared to baseline value.
